# Supplementary material for: A streamlined platform for analyzing tera-scale DDA and DIA mass spectrometry data enables highly sensitive immunopeptidomics
Source: Nat Commun. 2022 Jun 7;13:3108. doi: 10.1038/s41467-022-30867-7 (PMC9174175; doi:10.1038/s41467-022-30867-7)
Supplement: Supplementary file 1 — Supplementary Information [file 41467_2022_30867_MOESM1_ESM.pdf]

Supplementary Information:

An ultra-sensitive and streamlined platform for high-throughput analysis of tera-scale DDA and DIA mass spectrometry data and its applications in immunopeptidomics

B. Shan, M. Li et al.

Supplementary Note 1

Two different analyses in cancer immunopeptidomics were performed in this section. The first one was performed on three DDA immunopeptidomics datasets from Sarkizova et al. [1] and Bassani-Sternberg et al. [2]. The second one was performed on the immunopeptidomics sample RA957 from Pak et al. [3], which included both DDA and DIA data.

PEAKS Online was evaluated on three DDA immunopeptidomics datasets from Sarkizova et al. [1] and Bassani-Sternberg et al. [2] as following. The data was searched against a UniProt FASTA database of 42,148 entries (March 2017) with unspecific enzyme digestion. Methionine oxidation and protein N-terminal acetylation were used as variable modifications and no fixed modification was set. The precursor ion and fragment ion mass error tolerances were set to 10 ppm and 0.05 Dalton, respectively. The peptide FDR was first set at 1% to evaluate standard database search results of PEAKS Online versus MaxQuant (MaxQuant results were obtained from Sarkizova et al. [1] and Bassani-Sternberg et al. [2]). The peptide FDR was then set at 100% and all target and decoy PSMs were used for rescoring. We trained a deep learning-based spectrum prediction model for HLA peptides. Our prediction models can include three common variable modifications, including N-terminal (Acetylation), M(Oxidation), and NQ(Deamidation). In this manuscript, we started with some internal pre-trained models on tryptic peptides and re-trained them with HLA data from Sarkizova et al. [1]. For the evaluation in Figure 1f, the data of Mel-15, A*02:04, and A*02:06 were excluded from the training data. The predicted spectra were used to rescore the PSMs and the peptide FDR 1% was applied again after the rescoring. More details about the spectrum prediction model and the rescoring procedure were described above.

We also applied our platform to the immunopeptidomics sample RA957 published recently by Pak et al. [3]. DDA data was searched against a UniProt FASTA database of 42,148 entries (March 2017) with unspecific enzyme digestion. Methionine oxidation and protein N-terminal acetylation were used as variable modifications and no fixed modification was set. The precursor ion and fragment ion mass error tolerances were set to 6 ppm and 0.02 Dalton, respectively, together with 1% PSM FDR. Sample-specific spectral libraries were then generated from the DDA database search results. DIA data was searched against the generated spectral libraries with precursor ion and fragment ion mass error tolerances of 6 ppm

and 0.02 Dalton, respectively, and 1% peptide FDR. For DIA spectra with no library search results, DIA database search was performed with similar FASTA database, unspecific enzyme digestion, modifications, mass error tolerances, and FDR. Deep learning-based predicted spectra were used for peptides with no corresponding spectra available in the library. For DIA spectra with no library nor database search results, DIA de novo sequencing was performed. De novo peptides were filtered by applying a positional confidence score cutoff ALC >= 90. The list of peptides identified by the three computational approaches (spectral library search, database search, and de novo sequencing) were used to build a new spectral library. A final search of the whole dataset was performed against the new library with a unified global FDR of 1%.

Supplementary Note 2

We applied our platform to analyze the HLA-I immunopeptidome of SARS-CoV-2-infected A549 cells and HEK293T cells, which were published recently by Weingarten-Gabbay et al. [4]. A RefSeq-based sequence database was provided by Weingarten-Gabbay et al., which contains 41,457 proteins mapped to the human reference genome (hg38, June 29, 2018), 13 proteins encoded in the human mitochondrial genome, 264 common laboratory contaminant proteins, 553 human non-canonical small open reading frames, 27 SARS-CoV-2 proteins obtained from RefSeq derived from the original Wuhan-Hu-1 China isolate NC_045512.2, 2 SARS-CoV-2 proteins PODTD2 and PODTD3, and 23 novel unannotated virus ORFs whose translation is supported by Ribo-seq. There are in total 42,339 protein sequences in the database. PEAKS Online DB search engine was run with unspecific enzyme digestion. Cysteinylation of cysteine, methionine oxidation, deamidation of asparagine, protein N-terminal acetylation, and pyroglutamic acid at peptide N-terminal glutamine were used as variable modifications while no fixed modification was set. The precursor ion and fragment ion mass error tolerances were set to 10 ppm and 0.01 Dalton, respectively. The peptide length ranges from 7 to 20 amino acids. As in the previous study, peptide identifications were made by applying 1.5% FDR at the PSM level. Only 8- to 11-mer identified peptides were retained for all subsequent analyses, unless otherwise specified.

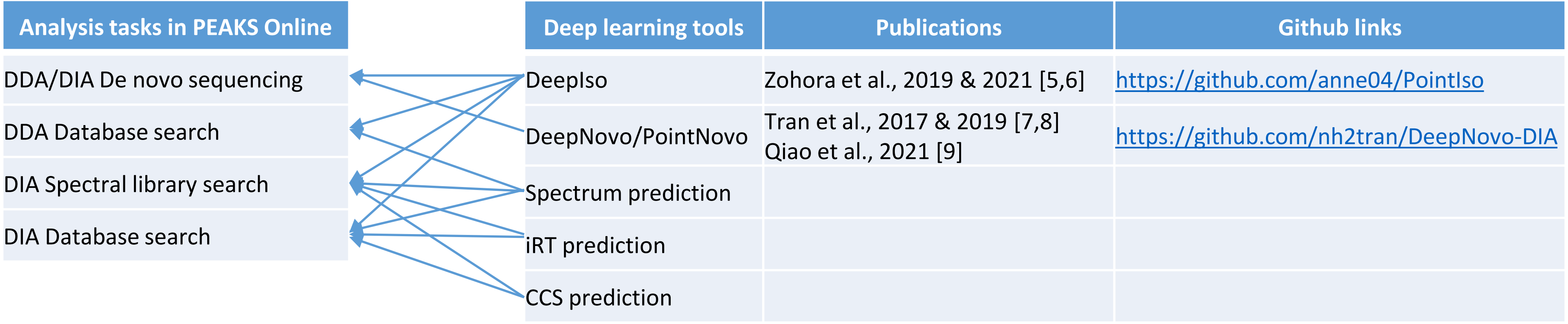

Supplementary Figure 1. Deep learning tools and their applications for different analysis tasks in PEAKS Online.

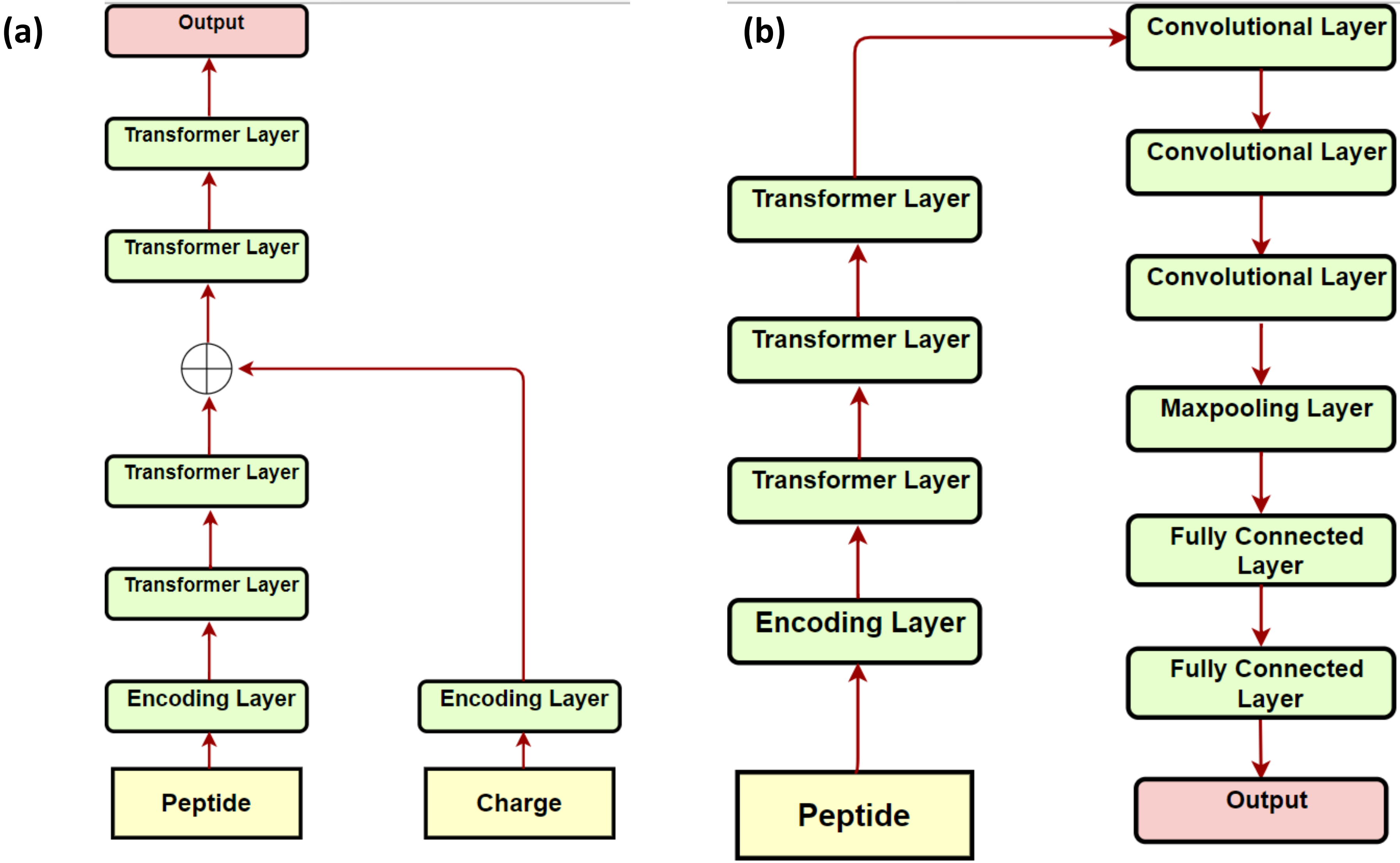

Supplementary Figure 2. Spectrum prediction model **(a)** and retention time prediction model **(b)** in PEAKS Online.

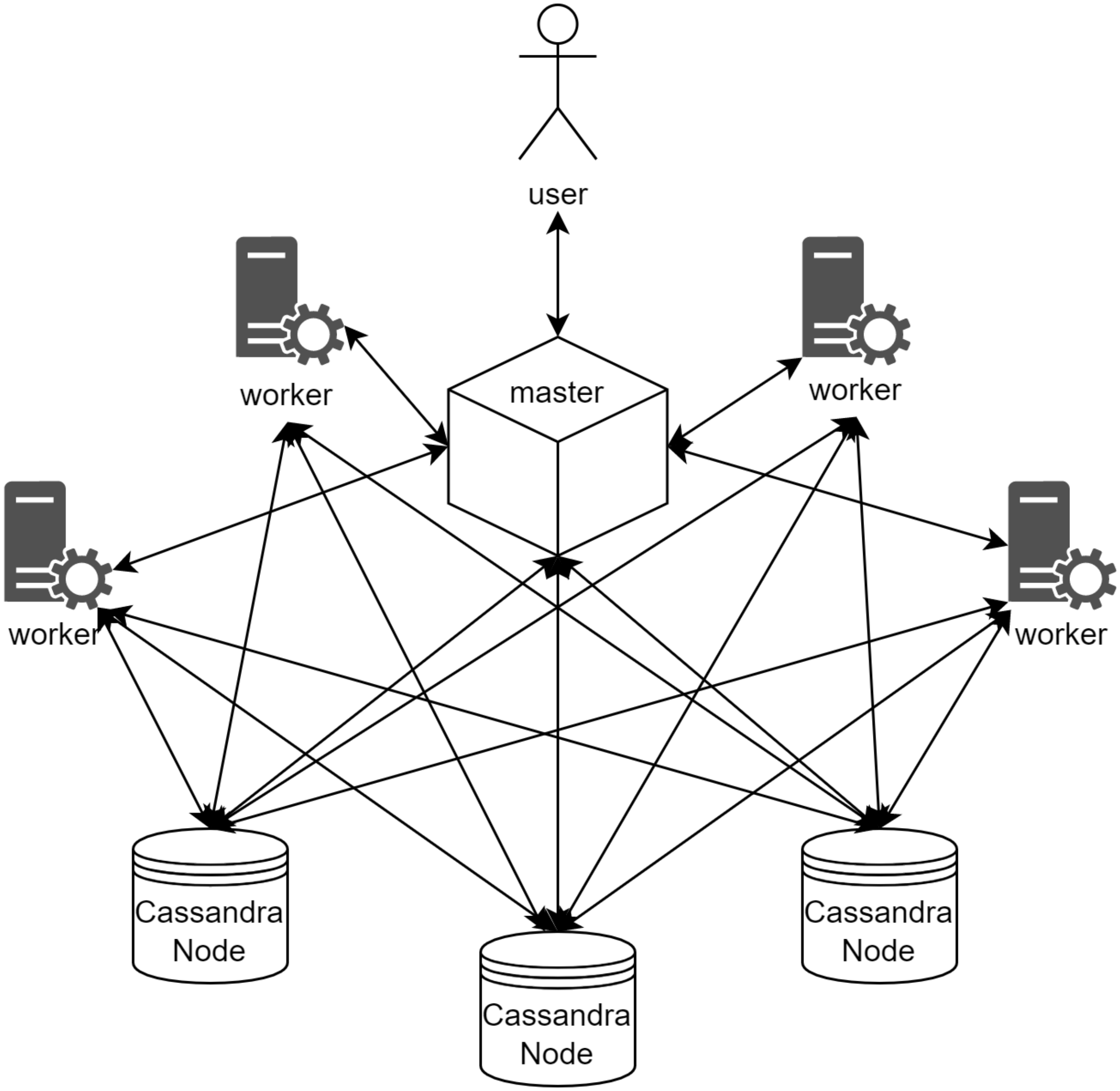

Supplementary Figure 3. The distributed high-performance computing architecture of PEAKS Online.

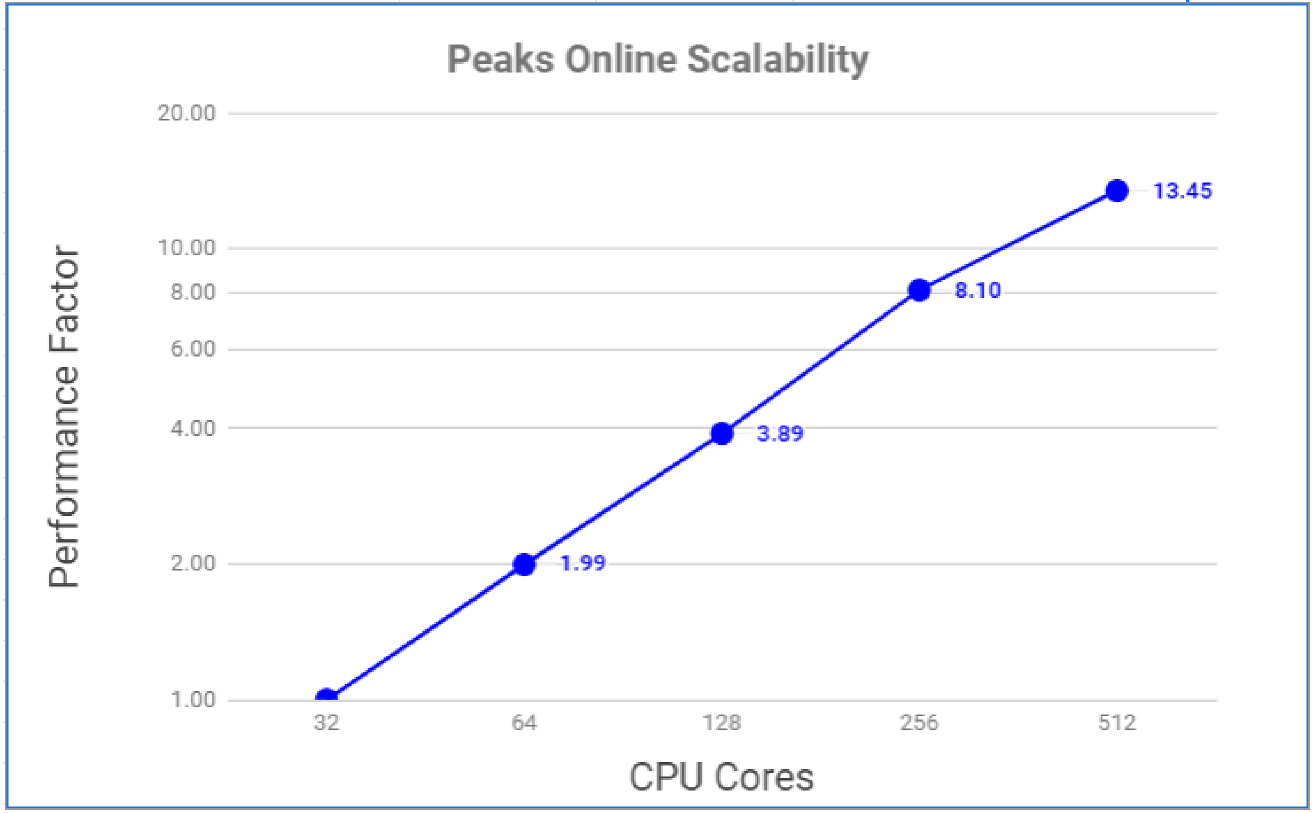

Total Data Size	1 TB
Total Samples	56
Total MS runs (180 mins each run)	672
MS	5,106,542
MS/MS	28,858,408
Time	CPU Cores
17h11m	512
1 day 4h32m	256
2 days 1h24m	128
4 days 19h55m	64
9 days 15h10m	32

Supplementary Figure 4. Example of PEAKS Online's scalability.

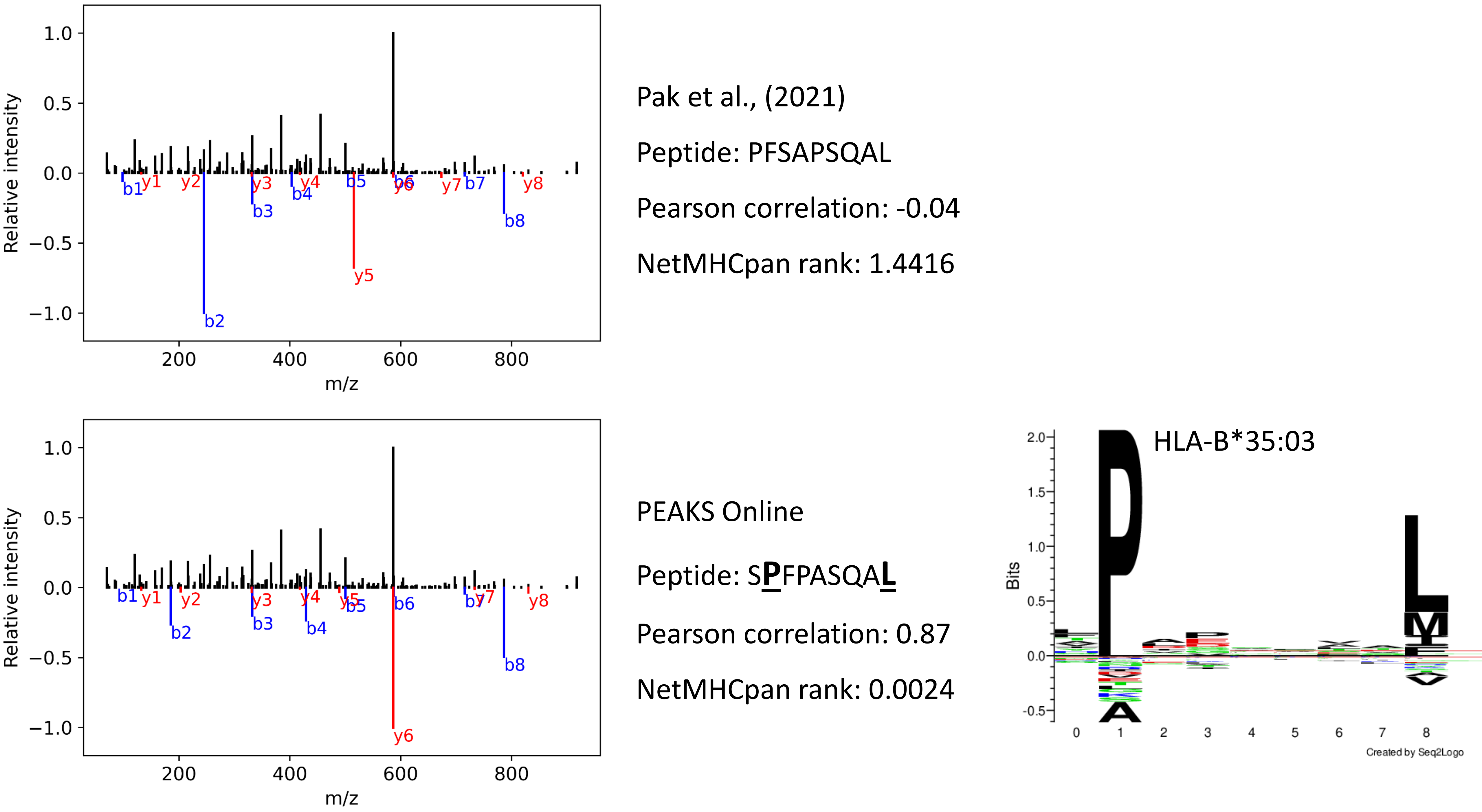

Supplementary Figure 5. Comparison of the peptides identified by Pak et al. [3] and PEAKS Online from the MS/MS scan id 57611, fraction RA957_R03. In the mirror plots, the top is the experimental spectrum, the bottom is the spectrum predicted by MS^{2}PIP from the corresponding peptide. The bottom-right panel shows the binding motif of HLA-B*35:03 from NetMHCpan. Overall, the Pearson correlation, the NetMHCpan rank and the binding motif indicate that the peptide identified by PEAKS Online is more accurate than the one by Pak et al.

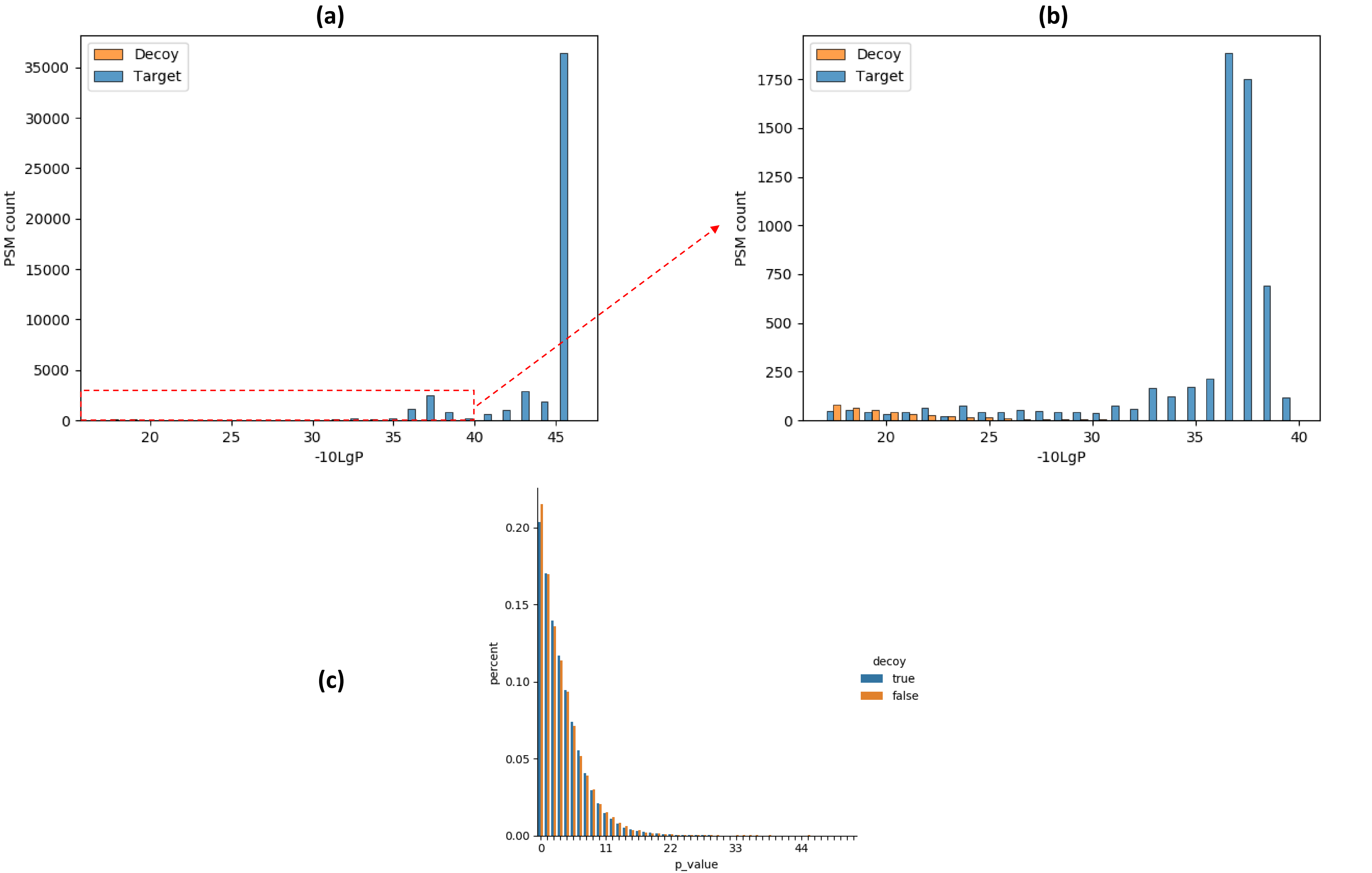

Supplementary Figure 6. The PSM score distributions of random decoy peptides and target peptides identified by PEAKS Online from the DIA dataset RA957 (Figure 3a). **(a, b)** As expected for FDR control, the score distribution of the random decoy peptides was indeed located at the lower end of the score distribution of the target peptides. **(c)** Decoy and target score distributions fully overlapped when the precursor masses were shifted by 100 Dalton. (PSM: peptide-spectrum match; FDR: false discovery rate).

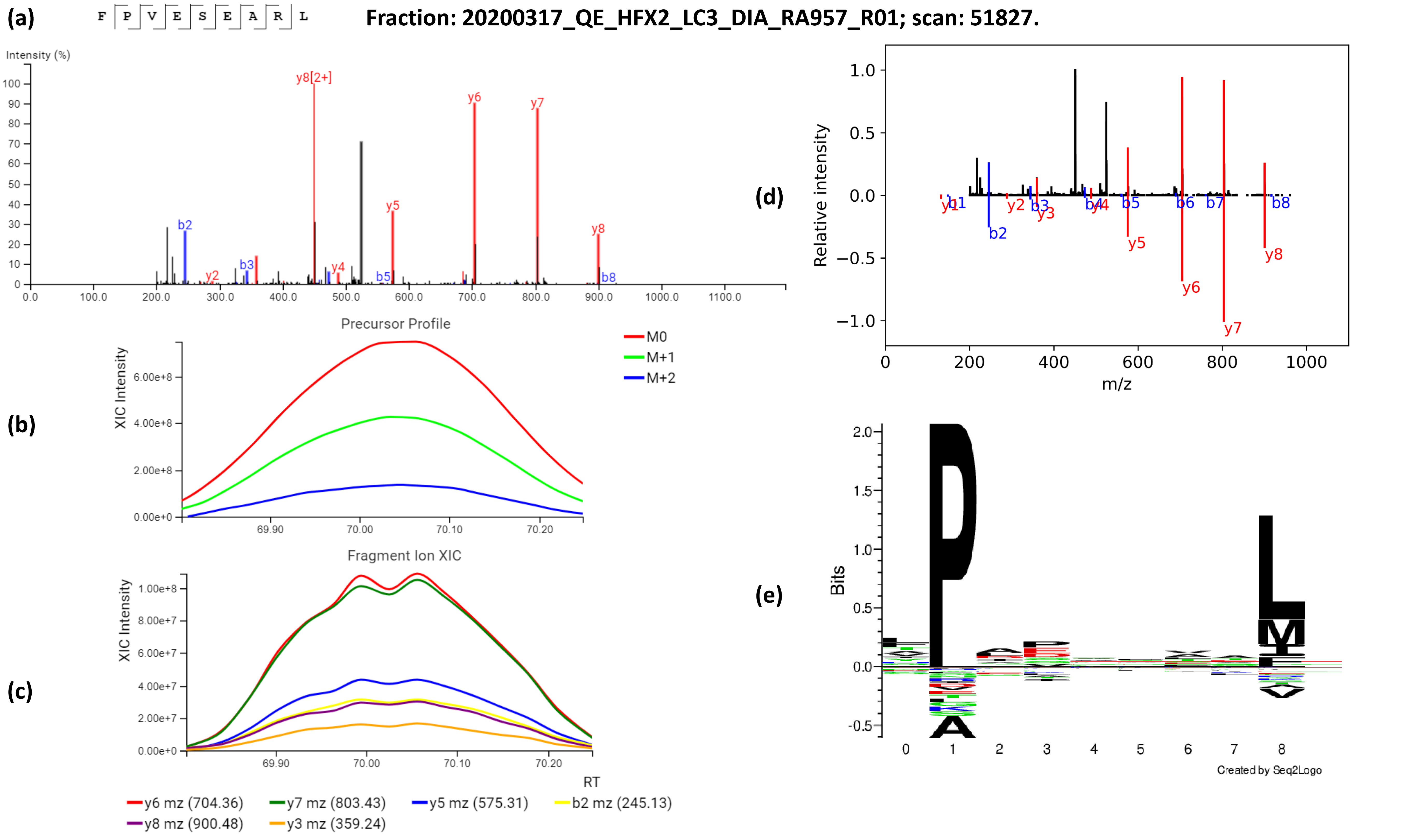

Supplementary Figure 7. Examples of de novo peptides identified by PEAKS Online from the DIA RA957 dataset. **(a)** Peptide-spectrum match. **(b)** Precursor profile. **(c)** Fragment ion profiles. **(d)** Mirror plot between the experimental spectrum and the predicted spectrum by MS2PIP. **(e)** Binding motif of the corresponding HLA allele.

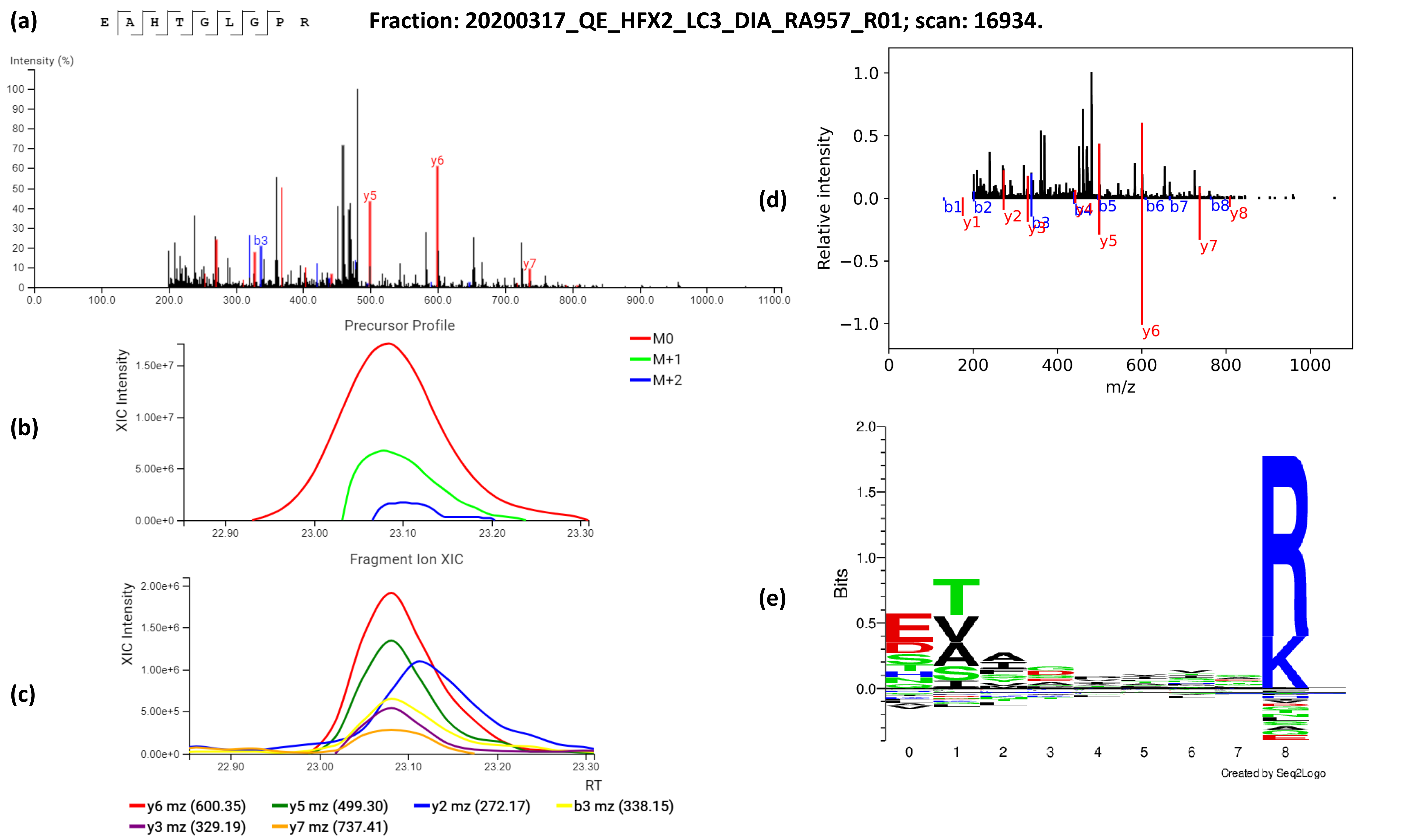

Supplementary Figure 8. Examples of de novo peptides identified by PEAKS Online from the DIA RA957 dataset. **(a)** Peptide-spectrum match. **(b)** Precursor profile. **(c)** Fragment ion profiles. **(d)** Mirror plot between the experimental spectrum and the predicted spectrum by MS2PIP. **(e)** Binding motif of the corresponding HLA allele.

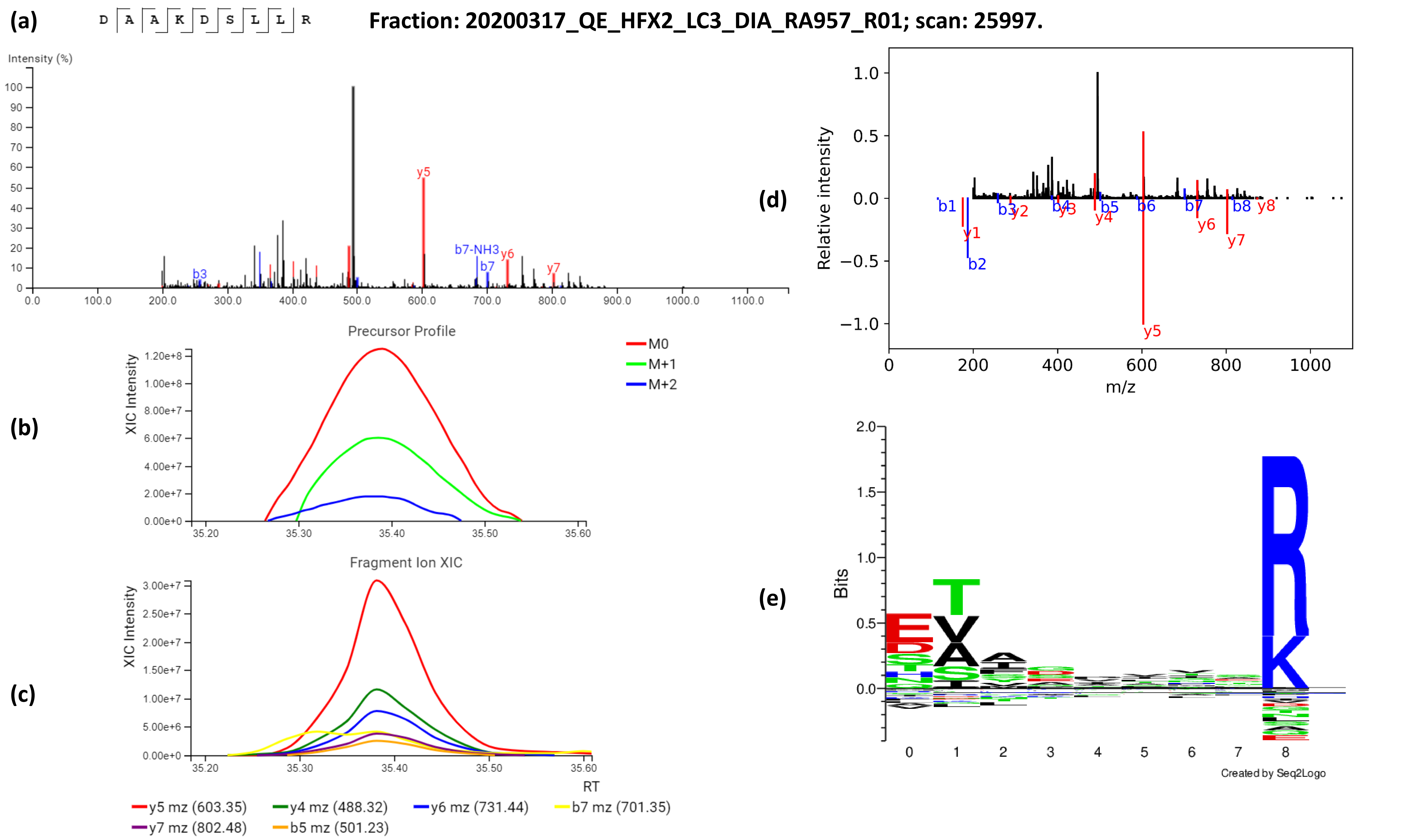

Supplementary Figure 9. Examples of de novo peptides identified by PEAKS Online from the DIA RA957 dataset. **(a)** Peptide-spectrum match. **(b)** Precursor profile. **(c)** Fragment ion profiles. **(d)** Mirror plot between the experimental spectrum and the predicted spectrum by MS2PIP. **(e)** Binding motif of the corresponding HLA allele.

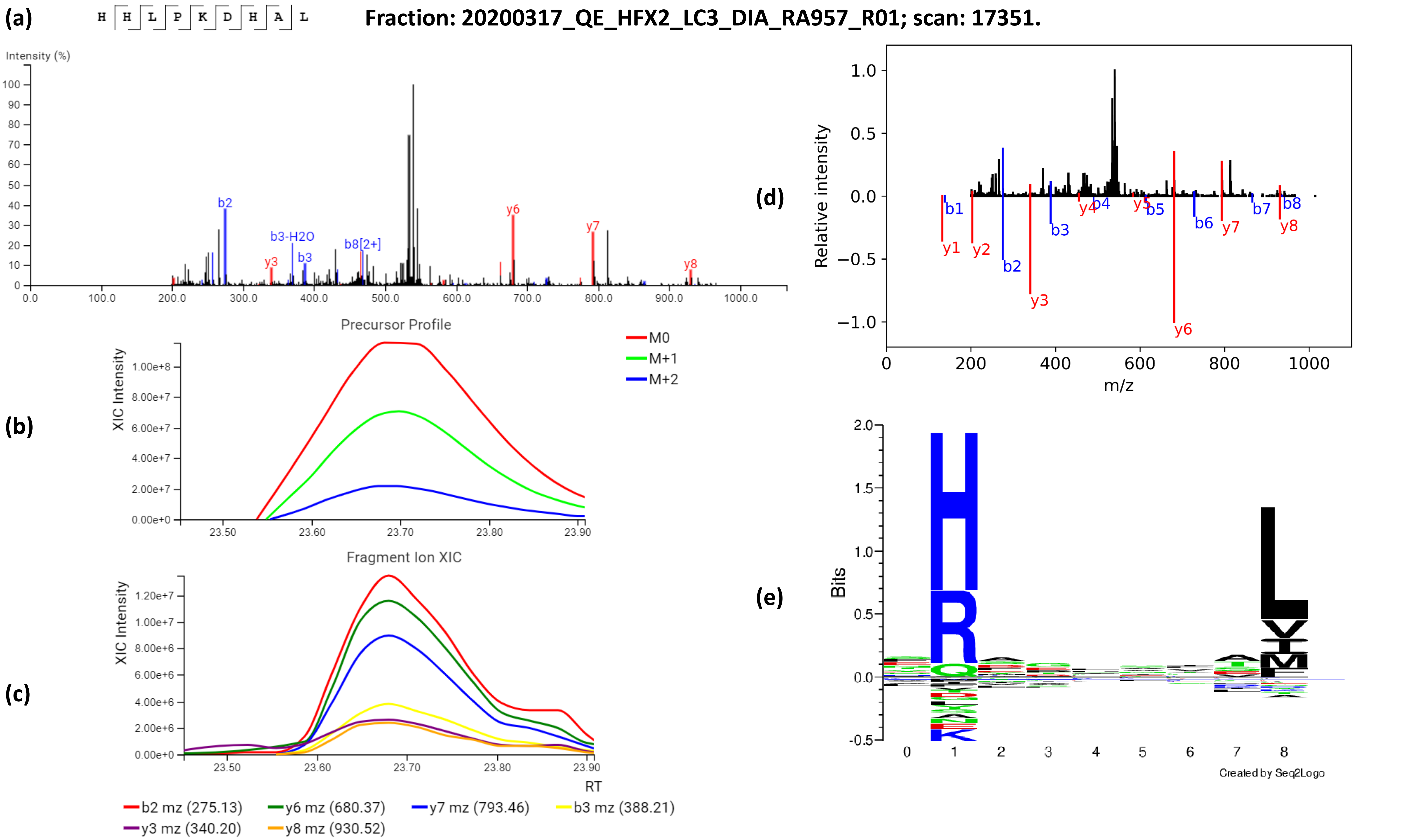

Supplementary Figure 10. Examples of de novo peptides identified by PEAKS Online from the DIA RA957 dataset. **(a)** Peptide-spectrum match. **(b)** Precursor profile. **(c)** Fragment ion profiles. **(d)** Mirror plot between the experimental spectrum and the predicted spectrum by MS2PIP. **(e)** Binding motif of the corresponding HLA allele.

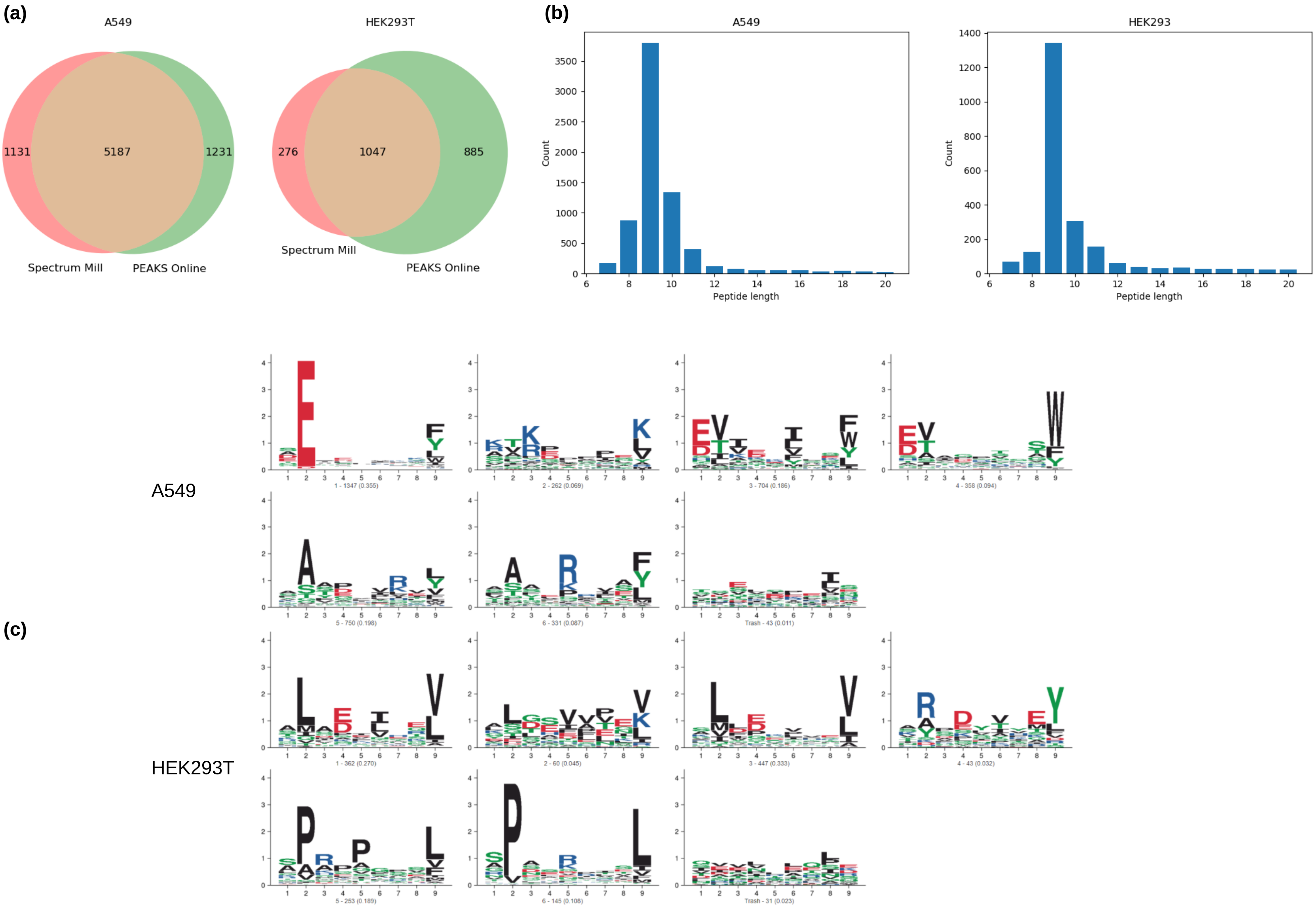

Supplementary Figure 11. Analysis of the HLA-I immunopeptidome of SARS-CoV-2-infected A549 cells and HEK293T cells. (a) Venn diagram of peptides identified by PEAKS Online and Spectrum Mill. **(b)** Length distribution of peptides identified by PEAKS Online. **(c)** Binding motif deconvolution of peptides identified by PEAKS Online.

(a)

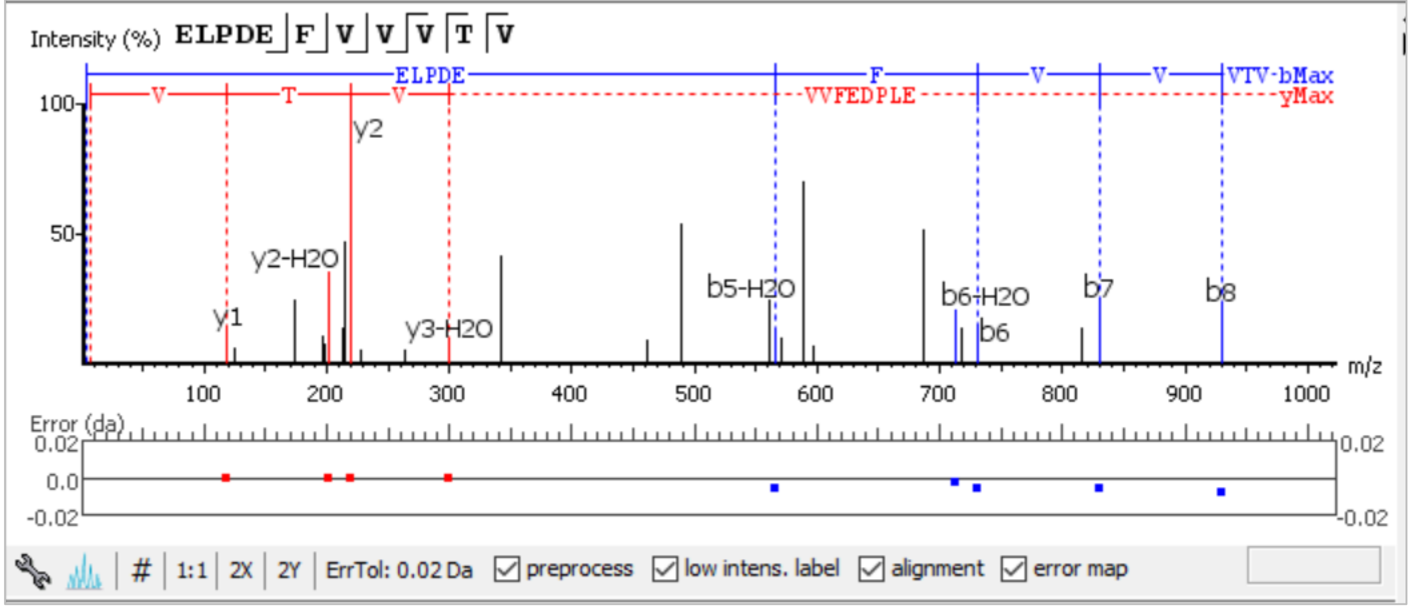

(b)

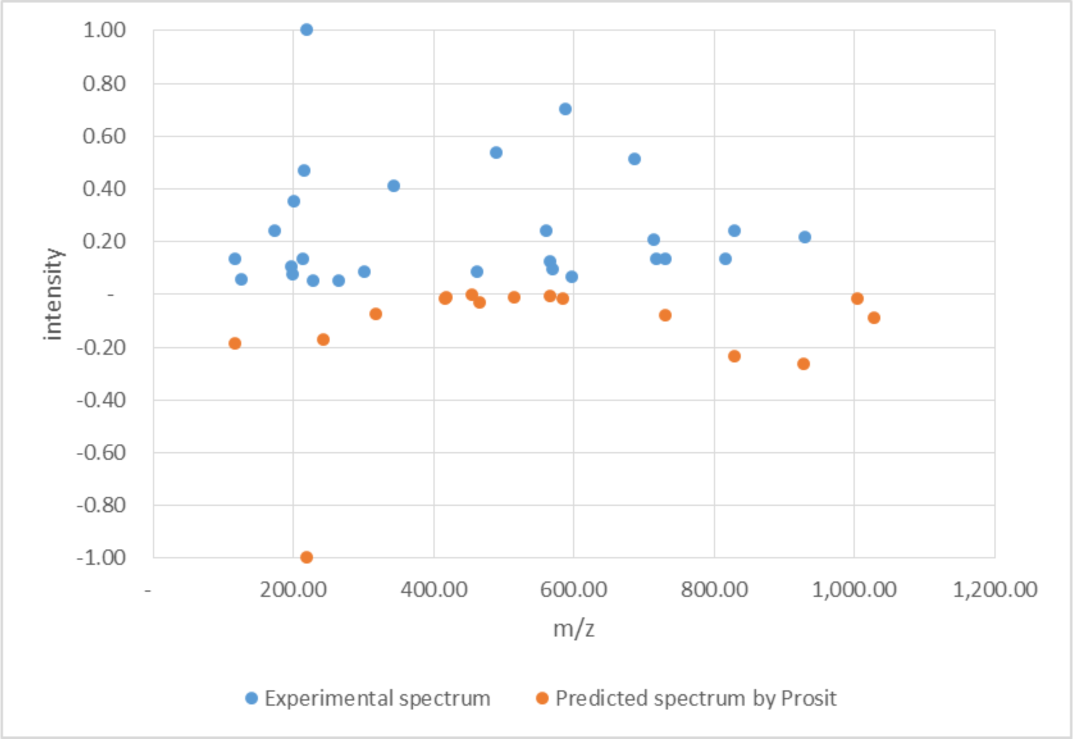

Supplementary Figure 12. Analysis of peptide ELPDEFVVVTV**. (a)** Peptide-spectrum match. **(b)** Experimental spectrum versus predicted spectrum by Prosit

Supplementary Table 1. List of features used for spectrum matching in PEAKS DIA.

1	absolute difference between observed RT and predicted RT
2	logarithm of the precursor ion intensity
3	number of co-eluted fragment ions
4	peaks raw score based on the dot product between library spectrum and observed spectrum
5	feature area
6	The std of absolute frag ions mass differences
7	The ratio between the summation of intensities of all matched frag ions and the summation of intensities of all peaks
8	charge
9	peptide length
10-16	the dot product score of the seven ms2 spectra around the apex RT
17-23	the spectra angle score of the seven ms2 spectra around the apex RT
24	the relative position of the RT in the whole experiment ((RT – RT_min) / (RT_max - RT_min))
25	the m/z of the precursor
26-34	The pearson correlation of the top 9 frag ions with the elution profile (coelution pattern)
35	the ratio between sum of the frag ion intensities and the precursor ion intensity
36	logarithm of the summation of frag ion intensities
37	logarithm of the summation of all corresponding ms1 precursor ion intensities

Supplementary Table 2. Example of a large project run on the distributed Amazon Web Services (AWS) cluster.

Total Data Size (GB)	3,683
Total Sample Number	4,407
Total MS Runs	5,397
Total Instrument Time (hours)	1,079
Time Upload to AWS (hours)	102
Total Processing Time on AWS (hours)	212
Number of CPU cores	512
RAM (TB)	1

Supplementary References

1. Sarkizova, S. *et al.* A large peptidome dataset improves HLA class I epitope prediction across most of the human population. *Nat. Biotechnol.* **38**, 199–209 (2020).
2. Bassani-Sternberg, M. *et al.* Direct identification of clinically relevant neoepitopes presented on native human melanoma tissue by mass spectrometry. *Nat. Commun.* **7**, 13404 (2016).
3. Pak, H. *et al.* Sensitive Immunopeptidomics by Leveraging Available Large-Scale Multi-HLA Spectral Libraries, Data-Independent Acquisition, and MS/MS Prediction. *Mol. Cell. Proteomics* **20**, 100080 (2021).
4. Weingarten-Gabbay, S. *et al.* Profiling SARS-CoV-2 HLA-I peptidome reveals T cell epitopes from out-of-frame ORFs. *Cell* **184**, 3962–3980.e17 (2021).
5. Zohora, F. T. *et al.* DeepIso: A Deep Learning Model for Peptide Feature Detection from LC-MS map. *Sci. Rep.* **9**, 17168 (2019).
6. Zohora, F. T. *et al.* Deep neural network for detecting arbitrary precision peptide features through attention based segmentation. *Sci. Rep.* **11**, 18249 (2021).
7. Tran, N. H. *et al.* Deep learning enables de novo peptide sequencing from data-independent-acquisition mass spectrometry. *Nat. Methods* **16**, 63–66 (2019).
8. Tran, N. H., Zhang, X., Xin, L., Shan, B. & Li, M. De novo peptide sequencing by deep learning. *Proc. Natl. Acad. Sci. U. S. A.* **114**, 8247–8252 (2017).
9. Qiao, R. *et al.* Computationally instrument-resolution-independent de novo peptide sequencing for high-resolution devices. *Nature Machine Intelligence* **3**, 420–425 (2021).
