# Supplementary material for: A streamlined platform for analyzing tera-scale DDA and DIA mass spectrometry data enables highly sensitive immunopeptidomics
Source: Nat Commun. 2022 Jun 7;13:3108. doi: 10.1038/s41467-022-30867-7 (PMC9174175; doi:10.1038/s41467-022-30867-7)
Supplement: Supplementary file 3 — Description of Additional Supplementary Files [file 41467_2022_30867_MOESM3_ESM.docx]

File Name: Supplementary Data 1

Description: Peptide-spectrum matches with different peptide identifications by Pak et al. [3] and PEAKS Online.

File Name: Supplementary Data 2

Description: List of all peptide-spectrum matches identified by PEAKS Online from the DIA dataset RA957.

File Name: Supplementary Data 3

Description: SARS-CoV-2 HLA-I peptides identified from infected A549 cells and HEK293T cells.
